# 

**Published:** 1887-07

**Authors:** 


### PORTRAIT ENGRAVINGS.

Dr. A. R. Kilpatrick, .......................... Deceased

Dr. S. F. Starley, .......................... Deceased.

Dr. T. D. Wooten, .......................... Austin.

Dr. J. F. Y. Paine, .......................... Galveston.

### CONTRIBUTORS TO VOL. III.

Drs. Odo Betz . . . Heilbron, Ger.
S. H. Condon ..... ... Arkansas
H. S. Robinson ........ "
C. M. Reynolds ....... "
E. J. Beall .......... Fort Worth
E. M. Bridgeford ...... Missouri
E. Meierhof ....... New York
T. J. Tyner ............. Austin
J. W. McLaughlin ...... "
A. N. Denton .......... "
Q. C. Smith ........... "
T. J. Bennett ......... "
C. O Weller ........... "
T. C. Osborn ......... Cleburne
J. L. Felder .......... "
T. J. Heard ......... Galveston
W. C. Fisher ......... "
A. F. Sampson ....... "
Wm. Caston .......... Corsicana
R. P. Davis ............... Petty
W. M. Powell ........... Albany
M. J. Birdsong ....... Greenville
F. E. Yoakum ....... "
J. E. Roach ....... Sipe Springs
Bat. Smith ........... Wharton
S. L. Post .......... Gainesville
W. M. Yandell .......... El Paso
C. M. Harrison .......... "
L. W. Cocke .. ... San Marcos
S. T. Lowry ....... San Antonio
B. F. Kingsley ..... "
R. Menger ........ San Antonio
R. H. Harrison, Jr. "
D. Berry .......... "
R. S. Gregg ............. Manor
J. H. Smith .......... Dallas
F. A. Schmitt . .. Schulenburg
W. W. Walker ..... "
E. T. Paine ......... Comanche
F. H. Tucker .... San Augustine
R. H. Day. .. Baton Rouge, La
G. W. Christian ........ Burnett
A. A. Lyon ...... Shreveport, La
R. B White ............ Ennis
A. J. Gray ....... Martin's Mills
L. M. Stroud .......... Forney
A. H. McCord ........... Rusk
W. M. Cunningham .... Bastrop
J. A Black ........ Round Rock
A. N. Perkins ...... Sabine Pass
J. E. Mayfield .... Nacogdoches
Q. A. Shuferd ........... Tyler
D. R. Wallace .......... Terrell
L. J Graham ..... Henderson
L. D. Hill ........ Webberville
R. P. Talley ............ Belton
J. T. Hutchins ........ Oakland
A. S. Wolff ......... Brownsville
E. P. Vellum .......... U. S. A.
E. N. Mendenhall ........ Plano
R. T. Knox ........... Gonzales
S. A. Stone .......... Richmond

